# Utilization of integrated HIV and sexual and reproductive health services among women in Uganda

**DOI:** 10.1186/s12913-016-1761-3

**Published:** 2016-09-20

**Authors:** Gideon Rutaremwa, Allen Kabagenyi

**Affiliations:** 1 United Nations Economic Commission for Africa (ECA), Social Development Policy Division, P.O.Box 3001, Addis Ababa, Ethiopia; 2 Center for Population and Applied Statistics (CPAS) and Department of Population Studies, Makerere University, Kampala, Uganda

**Keywords:** Utilization, Integrated, HIV, Sexual, Reproductive Health, Women and Uganda

## Abstract

**Background:**

While the rationale for integration of HIV and sexual and reproductive health (HIV and SRH) services is strong, there is paucity of information on which population groups most utilize these services. Such studies would inform policy and programs on integration of services. The overall objective of this assessment is to provide information to researchers, planners and policy makers on the best practices for integrated services in order to maximize feasibility of scaling up. Specifically, this research paper identifies demographic and socioeconomic factors that are most related to utilization of integrated services in Uganda.

**Methods:**

This manuscript uses data from a sample of 9,691 women interviewed during the Uganda AIDS Indicator Survey (UAIS) of 2011. The selection criteria of the study respondents for this paper included women of reproductive age 15 – 49 years. The dependent variable is whether the respondent utilized integrated HIV and SRH services during pregnancy and delivery of the last child, while independent variables include; region of residence, age-group of woman, marital status, rural-urban residence, wealth indicator and educational level attainment. In the main analysis, a binary logistic regression model was fitted to the data.

**Results:**

Log-odds of utilizing integrated services were significantly higher among those women with a primary education (OR = 1.2, 95 % CI = 1.0-1.4, *p* < 0.05) compared to those with no education. Women from the Central part of Uganda were more likely to utilize integrated HIV and SRH services (OR = 1.3, 95 % CI = 1.0-1.7, *p* < 0.05), further the log-odds of utilizing integrated HIV and SRH services were significantly higher among women residing in Northern region (OR = 1.6, 95 % CI = 1.2-2.2, *p* < 0.01). The odds of utilization of integrated HIV and SRH services were higher for currently married women (OR = 6.6, 95 % CI = 5.5-8.0, *p* < 0.01) and the formerly married (OR = 3.4, 95 % CI = 2.7-4.2, *p* < 0.01), compared to the never married group. The odds of utilizing integrated HIV and SRH services were higher for younger women of ages less than 35 years compared to older women aged 40 – 49 years.

**Conclusions:**

Utilization of integrated HIV and SRH services in Uganda is influenced greatly by demographic and socioeconomic characteristics. This study contributes to the current debate as it shows the on how best ways to improve HIV and SRH service delivery to the people.

## Background

Offering integrated HIV and Sexual and Reproductive Health (SRH) services is considered an effective means to manage and deliver care [1]. This is so because integration of SRH and HIV services has the potential to simultaneously address multiple patient needs in one location. For example, there has been broad international consensus that the goal of preventing mother-to-child transmission-PMTCT of HIV cannot be met without increasing access to family planning-FP services [2–4].

Whereas HIV and SRH services are related, and there are important potential benefits for both the client and health system associated with linking and integrating the provision of these services [1, 2, 5]. The rationale for utilization of integrated services is that there is potential for increase in the coverage of HIV services, as individuals who use SRH services can benefit from HIV services and vice-versa, as well as increase cost-savings [1, 6–8]. Within high HIV prevalence settings, the integration of HIV and SRH services has been widely regarded as beneficial in not only improving individual outcomes and reducing HIV transmission, but also improving the efficiency of service delivery [9]. In a study of integration opportunities in Addis Ababa Ethiopia, it was found that due to lack of integration, women were at risk of not receiving needed FP or HIV care services [10].

Despite the inadequate evidence on the procedure of operationalizing integrated programs, the dual family planning-HIV needs for clients are clear. Some studies have documented the levels of unmet need for family planning ranging from 14 to 67 % among clients of voluntary counselling and testing (VCT) [11], and from 9 % to 56 % among ART clients in Ghana [12]. Likewise, levels of unplanned pregnancies among HIV positive women appear to be much higher: from 51 % to 99 % of HIV positive women reported among studies in Côte d’Ivoire, South Africa, and Uganda [13–15]. A study in South Africa, examining the feasibility and acceptability of integrating counseling and testing services in family planning clinics, found that family planning clients were at risk of contracting HIV and other sexually transmitted infections (STIs). Although 17 % had multiple partners, 30 % knew their HIV status, and 40 % did not use a condom at last sex, while only 23 % of clients perceived a high risk of HIV [16]. This study further investigated the predictors of utilization of HIV and SRH services, the latter encompassing family planning services, prenatal, delivery care and post-delivery.

Admchack, and others [17], in their framework identified three models of integrated HIV and FP services. These were included in the study: family planning in HIV counseling and testing (FP-CT), family planning services in care and treatment services (FP-C&Tx), and HIV services (particularly CT) in family planning (HIV-FP). These models were selected a priori because they have the potential to: 1). Increase access to FP services for clients who do not use traditional FP services (FP-CT). 2). Increase uptake of contraception among women with HIV (FP-C&TX). 3) Increase access to HIV services such as CT and referrals for treatment among clients of reproductive age (HIV-FP).

The framework of Adamchack and others guides the choice of the outcome variable examined in this manuscript. Furthermore, the Andersen’s behavioral model of health care services utilization, which has been widely applied on survey data and various health services and populations [18–20], was used to guide selection of covariates that could affect utilization of both HIV and SRH services utilization.

Studies suggest that ANC service provision and the quality of services offered, are key components of healthcare delivery [21]. Evidence from health facilities three countries, namely Uganda, Tanzania and Burkina Faso suggest that health workers did not follow the recommended guidelines for health services delivery even when medical supplies were made available at the health facilities [22]. Furthermore, evidence from a Nepalese study suggest that a number of factors including: distance to the maternity hospital, low amenity score status, education, multi-parity, and antenatal care during pregnancy were associated with place of delivery by the mother [23, 24].

Whereas utilization of post-natal care services remains very low in many countries [24–26], PNC also appears to be related to the degree of awareness of availability of such services as well as to other factors including: level of awareness of the services, women’s occupation, ethnicity, the number of pregnancies and children, and the husbands’ socioeconomic status, occupation and education [25]. It is also possible that women experiencing postnatal health problems will always have a high propensity of seeking postnatal care compared to those who do not have such health complications. In addition, women who do not use ANC services would be less likely to utilize PNC. Finally, women who had a smooth delivery would be less likely to use PNC services compared to those who had prior complications during delivery.

In their study that focused on a systematic review of the Spaulding and others, suggest that combining family planning and HIV services was a feasible and effective intervention [27]. While the rationale for integration is strong [28–30], there is no firm evidence that suggests that integrated HIV and SRH services delivery result in increased contraceptive or HIV services utilization. Whereas the latter question may not be fully answered by the current study, it is important that the context of integrated HIV and SRH service delivery be clearly examined and for research evidence in this area to be developed. This paper seeks to explore the relationship between a number of predictor variables and utilization of integrated HIV and SRH services in Uganda. The reason for exploring these characteristics is that nearly all-existing studies have dwelt on benefits and effectiveness of offering integrated services. Understanding the characteristics of the clients is necessary for guiding programmes and elimination of inequality in access to services.

## Methods

### Data source

The 2011 Uganda AIDS Indicator Survey (UAIS) data were used for this study. Authorization to use the data was obtained from MEASURE DHS by providing a brief description of the study through their website. Permission to use the UAIS data for this study was obtained from ICF Macro International USA. Informed consent was obtained from all study participants at the time of data collection. Only eligible respondents who were willing to participate in the study were interviewed. The survey collected both background information as well as indicators on HIV and AIDS for all regions of Uganda, separate for urban and rural areas. The survey was conducted between February and September 2011 by the Uganda Ministry of Health [31].

### Explanatory variables

In this study, Andersen’s behavioral model of health service use was adapted to examine the relationship between individual-level socioeconomic and demographic factors and the utilization of a specific package of maternal health services. According to Andersen’s behavioral model, healthcare utilization is a function of three major elements: predisposing factors (socio-demographic factors), enabling factors (e.g. income and health insurance) and healthcare needs such as functional disability and chronic illnesses [18, 32, 33]. The predisposing factors in the model were: age, education level attainment, and region of residence, type of place of residence, religion, marital status and number of children ever born. Wealth indicator was considered an enabling factor, while visiting the health facility in the year prior to the survey was included in the model as a proxy for the need factor.

### Outcome variable

In this study the framework by Adamchack, and others [17] was adapted to derive the outcome variable. There are conceptual issues regarding the meaning of ‘integrated care’ and its measurement [34]. Whereas it may mean a combination of two previously separate components of care, it could also imply the addition of a new intervention dimensions into existing services [35]. In this manuscript therefore, integration is used to imply utilization of any of the HIV and SRH services jointly at a health service delivery facility during the pregnancy and delivery of the last child. The dependent variable was therefore dichotomous, that is, whether the respondent indicated that they had received any of the integrated HIV and SRH services. Women were asked a number of binary questions, which we combined to generate the variable “integrated HIV and SRH services”. The response variable was coded 1 if the individual answered affirmatively on any of the questions and zero otherwise. The following were the questions used:

1. Whether the individual woman sought STI advice from a family planning clinic.
2. Whether place of HIV test was family planning clinic.
3. Whether HIV testing was part of ANC visit
4. Whether individual got HIV test results as part of ANC visit.
5. Whether at last ANC visit individual talked about HIV; prevention of HIV and about getting a test for HIV.

### Sampling

This study utilized data from a sample of 9,691 women of ages 15–49 years, who were interviewed during the 2011 UAIS [36]. The 2011 UAIS collected information from over 11,000 households including 22,000 women of ages 15–49 years and men aged 15–59. Using the 2002 Uganda population and housing census sampling frame, a two stage sampling design was adopted, in which the first stage involved selecting 470 clusters (79 urban and 391 rural from a list of enumeration areas. At the second stage of sampling, 25 households were selected from each of the 470 clusters identified in the first stage. The inclusion criterion for survey participants was all individuals (men and women) age 15–59 years present in the household the night before the survey. Furthermore, blood sample testing for HIV was done on a voluntary basis for all survey respondents. Blood samples were also collected from children under age 5 with the consent of their parents or caretakers. Additional explanation on the study design and procedures for data collection are available in the main UAIS report [31].

### Statistical analyses

Univariate and bivariate analyses were done in order to describe the characteristics of women and their utilization of integrated HIV and SRH services. All explanatory variables with significant difference (*p* ≤ 0.05) were chosen and incorporated in the multivariate analyses. In the main analyses, a binary logit model was fitted to the data in order to predict whether individual woman utilized integrated HIV and SRH services or not. The odds ratios (ORs) based on a 95 % confidence interval (CI) were estimated for each of the categories of the independent covariates (Table 3). The regression model used is presented below:

1 $$ \mathrm{logit}\left[P\left(Y=1\right)\right]={\beta}_0+{\displaystyle \sum_{j=1}^k{\beta}_j{X}_j} $$

Where *logit [P(Y = 1)]* refers to the natural log-odds that a woman utilized integrated HIV and SRH services; β_0_ is the intercept of the equation fitted; and *β*_*j*_*X*_*j*_ are the regression estimates for a set of independent covariates (numbered 1 through k) contained in the model.

For purposes of accounting for the complex sample design used in the UAIS, we weighted the data during the analysis. The latter procedure was intended to account for the effects of clustering and stratification as well as the sampling weights when computing the variance, standard error and confidence intervals (the ***svyset*** option was used). The variables included in the regression model were checked for multi-collinearity before being added to the analysis model. Finally all analysis was conducted using STATA data analysis software version 13.

## Results

### Descriptive results

Table 1 presents the descriptive findings of our study. According to the results the percentage of respondents increased from 12 % for age group 15–19 to 21 % for the next category 20–24 years. More than 52 % of the study population was in the ages below 25 years, while only about 18 % were in the ages above 40 years. The findings also show that 79 % of the respondents in the study sample were resident in rural areas, while nearly 16 % had never attended school. Only about one quarter (24 %) had attained at least a secondary education level. Furthermore, 73 % of the respondents were currently married, together with those previously married, those ever married comprised nearly 90 % of the study population A considerable percentage (36.5 %) of the women was poor, while 45 % belonged to top two wealth quintiles (richer category). In terms of religious affiliation, 87 % of the study sample was Christian while Moslems formed only 8.5 % of the study sample. Regarding region of residence, Western region had a fairly bigger sample representation compared to all other regions (25.2 %), followed by Northern region (24.9 %), and Kampala region had the least representation (11 %). Finally, Table 1 also shows that nearly 59 % of the respondents indicated that they had received integrated HIV and SRH services at their last visit to a health facility.

**Table 1:** Weighted percentage distribution of respondents by selected background characteristics (*n* = 9720)

Variable/category	Number	Percent
Age group		
15-19	1,162	12.0
20-24	2,041	21.0
25-29	1,928	19.8
30-34	1,472	15.1
35-39	1,353	13.9
40-44	948	9.8
45-49	816	8.4
Rural/urban residence		
Urban	2,008	20.7
Rural	7,712	79.3
Education level		
None	1,535	15.8
Primary	5,829	60.0
At least secondary	2,356	24.2
Marital status		
Never married	1,202	12.4
Currently married	7,097	73.0
Previously married	1,422	14.6
Wealth indicator		
Poor	3,545	36.5
Middle	1,772	18.2
Richer	4,403	45.3
Religion		
Catholic	3,951	40.6
Protestant	4,506	46.4
Moslem	822	8.5
Other	441	4.5
Region of residence		
Kampala	1,073	11.0
Central	1,754	18.1
Eastern	2,019	20.8
Northern	2,422	24.9
Western	2,453	25.2
Received integrated services		
No	3,992	41.1
Yes	5,728	58.9

Table 2 shows the percentage distribution of women aged 15–49 years by their use of integrated HIV and SRH services. There was a strong association between utilization of integrated HIV and SRH services and women’s background characteristics, with exception of religious affiliation. Consequently we dropped religious affiliation from the multivariate logistic regression model due to non-significance at the bivariate stage of analysis.

**Table 2:** Distribution of women respondents by selected explanatory variables and by utilization of integrated HIV and SRH services (*n* = 9691)

Variable/category	Utilization of integrated HIV and SRH services		Pearson χ^2^	Significance
	No	Yes		
Age group				
15-19	16.9	8.3		
20-24	14.9	25.3		
25-29	10.8	25.8		
30-34	10.8	18.0	1400.0	*p* = 0.00
35-39	14.3	13.8		
40-44	14.9	6.2		
45-49	17.4	2.6		
Rural/urban residence				
Urban	23.1	18.7	27.6	*p* = 0.00
Rural	76.9	81.3		
Education level				
None	18.9	14.9		
Primary	55.3	62.6	54.4	*p* = 0.00
At least secondary	25.9	22.6		
Marital status				
Never married	21.5	5.4		
Currently married	59.1	82.8	774.2	*p* = 0.00
Previously married	19.4	11.8		
Wealth indicator				
Poor	35.0	41.5		
Middle	17.2	18.1	56.9	*p* = 0.00
Richer	47.8	40.3		
Religion				
Catholic	41.4	42.3		
Protestant	46.1	45.5	1.0	*p* = 0.80
Moslem	8.3	8.0		
Other	4.2	4.2		
Region of residence				
Kampala	9.3	9.3		
Central	21.7	19.0		
Eastern	24.0	18.7	100.3	*p* = 0.00
Northern	24.3	32.9		
Western	20.7	20.1		

### Multivariate results

The findings of the logistic regression equation fitted to predict the log-odds for utilizing integrated services are presented in Table 3. Three models were estimated (Table 3), and the results for the three models are presented in this section of the manuscript.

**Table 3:** Logistic regression model predicting the odds of utilizing integrated HIV and SRH services by women in Uganda

Variable/category	Model 1 (combined)		Model 2 (Rural)		Model 3 (Urban)	
	Odds ratio	95 % CI	Odds ratio	95 % CI	Odds ratio	95 % CI
Rural/urban residence						
Urban ^rc^	1.000	-	-	-	-	-
Rural	1.118	[0.9-1.3]	-	-	-	-
Age group						
15-19 ^rc^	1.000	-	-	-	-	-
20-24	***2.130	[1.8-2.6]	***2.039	[1.7-2.5]	***2.43	[1.5-4.0]
25-29	***2.526	[2.1-3.1]	***2.434	[2.0-3.0]	***2.87	[1.7-4.8]
30-34	***1.521	[1.2-1.9]	***1.514	[1.2-1.9]	1.55	[0.9-2.7]
35-39	0.874	[0.7-1.1]	0.943	[0.8-1.2]	*0.62	[0.4-1.1]
40-44	***0.375	[0.3-0.5]	***0.401	[0.3-0.5]	***0.25	[0.1-0.5]
45-49	***0.146	[0.1-0.2]	***0.156	[0.1-0.2]	***0.08	[0.0-0.2]
Education level						
None ^rc^	1.000	-	-	-	-	-
Primary	**1.220	[1.0-1.4]	**1.222	[1.0-1.4]	1.49	[0.9-2.4]
At least secondary	*1.190	[1.0-1.4]	1.194	[1.0-1.5]	1.43	[0.9-2.3]
Marital status						
Never married ^rc^	1.000	-	-	-	-	-
Currently married	***6.613	[5.5-8.0]	***5.785	[4.6-7.2]	***9.01	6.3-13.0]
Previously married	***3.369	[2.7-4.2]	***3.035	[2.4-3.9]	***4.27	2.8-6.6]
Wealth indicator						
Poor ^rc^	1.000	-	-	-	-	-
Middle	0.982	[0.8-1.1]	0.982	[0.8-1.1]	1.04	[0.3-4.2]
Richer	0.921	[0.8-1.1]	0.960	[0.8-1.1]	0.41	[0.1-1.3]
Region of residence^a^						
Kampala ^rc^	1.000	-	-	-	-	-
Central	**1.337	[1.0-1.7]	-	-	**1.56	[1.1-2.2]
Eastern	0.895	[0.7-1.1]	***0.699	[0.6-0.8]	0.96	[0.7-1.4]
Northern	***1.572	[1.2-2.0]	***1.302	[1.1-1.6]	0.92	[0.6-1.4]
Western	1.163	[0.9-1.5]	0.907	[0.8-1.1]	1.20	[0.8-1.8]
Model constant	***0.186	[0.1-0.3]	***0.289	[0.2-0.4]	**0.28	[0.1-1.0]
*Number of cases*	*9691*		*7707*		*1984*	
*F-statistic*	*82.1*		*73.1*		*18.1*	
*Model significance (p)*	*0.000*		*0.000*		*0.000*	

Note

*** significant at *p* < 1 %; ** significant at *p* < 5 %; * significant at *p* < 10 %

^**a**^ For the variable region of residence in model 1 (combined) Kampala is reference category, while in model 2 (rural) Central region is the reference category

*rc* reference category

The findings in Model 1 show that age, rural/urban residence, education, marital status and region of residence were significantly associated with utilization of integrated HIV and SRH services. The results further show that the odds of utilizing integrated HIV and SRH services were higher in the age groups 20–24 through 30–34 years compared to the reference category (age group 15–19). The pattern of age coefficients from the logistic regression model also show that the log-odds were significantly lower than the reference category for the age groups40–44 and 45–49. Women in the age group 25–29 has 2.5 times (OR = 2.5; 95 % CI = 2.1-3.1; *p* < 0.01) the odds of utilizing integrated HIV and SRH services compared to those in the age group 15–19, while women in the age group 45–49 had 0.15 times (OR = 0.1; 95 % CI = 0.1-0.2; *p* < 0.01) the odds of utilizing integrated HIV and SRH services compare to those in the reference category.

Concerning geographical region of residence, utilization of integrated HIV and SRH services was significantly higher in Central region of Uganda compared to Kampala the reference category (OR = 1.3; 95 % CI = 1.0-1.7; *p* < 0.01). Furthermore utilization of integrated services was highest in northern region of Uganda (OR = 1.6; 95 % CI = 1.2-2.0; *p* < 0.01).

Education of respondent was significantly associated with the level of utilization of integrated HIV and SRH services. The log-odds of utilizing integrated services were significantly higher among those women with a primary (OR = 1.2; 95 % CI = 1.0-1.4; *p* < 0.05), while those women with a secondary and higher level of education did not show any significant difference compared to those with no education, respectively. Finally, the log odds of utilizing integrated HIV and SRH services were significantly higher among the currently married (OR = 6.6; 95 % CI = 5.5-8.0; *p* < 0.01) and also among the formerly married women (OR = 3.4; 95 % CI = 2.7-4.2; *p* < 0.01) categories, compared to the never married group of women. The variables wealth indicator and rural-urban residence were not significant in the regression models.

The findings presented in Model 2 follow a similar pattern as in Model 1 for all the variables included in the regressions. It is important to note that the effect of education on utilization of integrated HIV and SRH services was significant in rural areas, and this effect disappears in and was not significant in Model 3 for urban areas. The wealth indicator variable was not significant in all the three models estimated. The three models showed varied odds ratios for the region of residence variable.

## Discussion

This study examined the relationship between some selected socioeconomic factors and utilization of integrated HIV and SRH services in Uganda. We observed that utilization of HIV and SRH integrated services in Uganda varied significantly by demographic and socioeconomic characteristics. It is therefore important to understand these differentials in order to ensure that policy and program efforts are targeted toward the groups that are most in need. The findings from this study show that women in Central and Northern Uganda were more likely to use integrated HIV and SRH services compared to those in Kampala region. Regional differences in utilization of HIV and SRH services could perhaps be explained by the differences in access to health services, the varying socio-cultural contexts and economic opportunities available to women in the respective regions of the country [37].

The variations in the findings of region of residence variable for the three models estimated can be explained by the fact that Kampala region is classified as 100 % urban and therefore was excluded from model 2 as a category. Rather Eastern region was used as a reference category in Model 2. Because of the latter reason, comparison of results of the three Models may not be appropriate.

Our analyses suggest that the age of the woman was significantly associated with the chances of utilization of integrated HIV and SRH services. The findings showed that younger women less than 35 years were more likely to utilize integrated HIV and SRH services compared to those in ages above 35 years. The latter finding could invariably be attributed to the age-varying integrated HIV and SRH services needs of women as the go through their reproductive life span. Therefore, higher levels of utilization of such integrated services tend to be in the lower ages as opposed to the higher ages after age 35. The implication for this finding is that whereas programs aimed at provision of integrated HIV and SRH services should target younger women, given that their effective demand for integrated HIV and SRH services is higher, addressing the needs of older women could prove effective in positively influencing HIV and SRH service provision in the country, given that women in Uganda continue to produce children even in ages 35–49 years.

The current findings indicate an increased level of utilization of integrated services among women with a primary level of education compared to those with no education. Attainment of a secondary and higher education level did not seem to increase the levels of utilization of integrated HIV and SRH services in Uganda. The latter finding suggests that the pattern of utilization of integrated HIV and SRH services is different from utilization of maternal health services generally, where secondary and higher education substantially increased women’s utilization of services [19, 37–39]. The possible explanation for this finding is that women who have a higher education tend to be wealthier and can afford the stand-alone services. It is therefore important that any policies and programs targeting improved utilization of services should be sensitive to educational level attainment of individuals.

Finally, as expected women who were ever married were more likely to use integrated HIV and SRH services compared to single women. This is because ever-married women tend to me more exposed to sexual encounter and therefore their need for both HIV and SRH services tends to be higher compared to that of single women.

### Study Limitations

The current study was based on cross-sectional individual level data and does not address health systems and community related factors that are known to influence health services utilization. Other limitations of this study relate to self-report measures that are often unreliable. This implies that information was self-reported which comes with respondent bias characteristic of cross-sectional surveys. Perhaps most importantly the study does not establish whether utilization of integrated HIV and SRH services improves overall utilization of HIV and SRH services, thereby improving the attendant health outcomes. The authors generated the measurement of the outcome variable therefore could have some limitations and could influenced the results. Despite these limitations, the current study utilized reliable data and appropriate analysis methods. The sample is representative of the whole country given the rigorous survey design and sample selection procedures. Consequently, the findings of this study reflect accurately on the relationship between selected socioeconomic factors and utilization of integrated HIV and SRH services.

## Conclusion

Given the intricate background in which HIV and SRH services are manifest in Uganda, it is important to understand and interpret these programs within their demographic and socioeconomic contexts, hence the relevance of this study. The nature and scope of the HIV epidemic, and the populations most affected within a country, determine the range and distribution of health services offered. Regional and local health policies, as well as program operational procedures, can facilitate or impede health service delivery. Strong reproductive health programs may be better positioned to incorporate new HIV-related services than weaker programs hampered by systemic resource constraints. Therefore, this paper contributes to the current debate on how best to improve HIV and SRH service delivery to the people in need and to the population of Uganda in particular. In order to harness the benefits of utilization of HIV and SRH services offered in an integrated manner, these intervention programs should target the geographic regions of the country that are less likely to utilize the latter services such as the urban areas. Education, age of woman and marital status are also important considerations for effective HIV and SRH integrated service delivery.

## Abbreviations

ANC: Antenatal care
ART: Anti-Retroviral Therapy
CDC: Centers for disease control and prevention
CI: Confidence interval
DHS: Demographic and health survey
FP: Family planning
FP-C&Tx: Family planning services in care and treatment services
FP-CT: Family planning in HIV counseling and testing
PMTCT: Prevention of mother- to- child transmission
SRH: Sexual and reproductive health
STIs: Sexually transmitted infections
UAIS: Uganda AIDS indicator survey
UBOS: Uganda bureau of statistics
UVRI: Uganda virus research institute
VCT: Voluntary COUNSELING AND TESting
